# Organ Preservation after Endoscopic Resection of Early Esophageal Cancer with a High Risk of Lymph Node Involvement

**DOI:** 10.3390/cancers12123598

**Published:** 2020-12-02

**Authors:** Solène Dermine, Thomas Lévi-Strauss, Einas Abou Ali, Arthur Belle, Sarah Leblanc, Jean-Emmanuel Bibault, Amélie Barré, Lola-Jade Palmieri, Catherine Brezault, Marion Dhooge, Benoit Terris, Anthony Dohan, Philippe Soyer, Arthur Berger, Gabriel Rahmi, Romain Coriat, Stanislas Chaussade, Maximilien Barret

**Affiliations:** 1 Department of Gastroenterology and Digestive Oncology, Cochin Hospital, Assistance Publique-Hôpitaux de Paris, 75014 Paris, France; t.levistrauss@yahoo.fr (T.L.-S.); einas.abouali@aphp.fr (E.A.A.); arthur.belle@aphp.fr (A.B.); sarahleblanc34@hotmail.com (S.L.); amelie.barre@aphp.fr (A.B.); lolajade.palmieri@aphp.fr (L.-J.P.); catherine.brezault@aphp.fr (C.B.); marion.dhooge@aphp.fr (M.D.); romain.coriat@aphp.fr (R.C.); stanislas.chaussade@aphp.fr (S.C.); maximilien.barret@aphp.fr (M.B.); 2 Department of Medecine, Université de Paris, 75006 Paris, France; jean-emmanuel.bibault@aphp.fr (J.-E.B.); benoit.terris@aphp.fr (B.T.); Anthony.dohan@aphp.fr (A.D.); philippe.soyer@aphp.fr (P.S.); arthur.berger.bx@gmail.com (A.B.); gabriel.rahmi@aphp.fr (G.R.); 3 Department of Radiation Oncology, Georges Pompidou European Hospital, Assistance Publique-Hôpitaux de Paris, 75015 Paris, France; 4 Department of Pathology, Cochin Hospital, Assistance Publique-Hôpitaux de Paris, 75014 Paris, France; 5 Department of Radiology, Cochin Hospital, Assistance Publique-Hôpitaux de Paris, 75014 Paris, France; 6 Department of Gastroenterology, Georges Pompidou European Hospital, Assistance Publique-Hôpitaux de Paris, 75015 Paris, France

**Keywords:** superficial esophageal cancer, endoscopic mucosal resection, endoscopic submucosal dissection, chemoradiotherapy, close follow-up, organ preservation

## Abstract

**Simple Summary:**

Virtually all early (T1) esophageal cancers can be resected endoscopically. However, the presence of histologic criteria on the resection specimen (deep submucosal invasion, lymphovascular involvement, poor tumor differentiation) are believed to be associated with a high risk (> 10%) of lymph node involvement. Therefore, the presence of such histoprognostic criteria currently require an esophagectomy. However, some patients are unfit for surgery or decline surgery, and undergo close follow-up or chemoradiotherapy. We analyzed the outcomes of these patients. We included 41 patients, of which thirteen (32%) were closely monitored, and 28 (68%) were treated by chemoradiotherapy or radiotherapy alone. After a mean follow-up of 19 and 28 months, cancer specific survival was 100% and 96%, respectively. Our study shows that close follow-up may be an alternative to esophagectomy after endoscopic resection of an early esophageal cancer with a predicted high risk of lymph node involvement.

**Abstract:**

*Background*: Esophagectomy is recommended after endoscopic resection of an early esophageal cancer when pejorative histoprognostic criteria indicate a high risk of lymph node involvement. Our aim was to analyze the clinical outcomes of a non-surgical, organ preserving management in this clinical setting. *Patients and Methods*: This retrospective study was performed in two tertiary centers from 2015 to 2020. Patients were included if they had histologically complete resection of an early esophageal cancer, with poor differentiation, lymphovascular invasion or deep submucosal invasion. Endoscopic resection was followed by chemoradiotherapy or follow-up in case of surgical contraindications or patient refusal. Outcome measures were disease-free survival (DFS), overall survival (OS), cancer specific survival (CSS) and toxicity of chemoradiotherapy. *Results*: Forty-one patients (36 with squamous cell carcinoma and 5 with adenocarcinomas) were included. The estimated high risk of lymph node involvement was based on poor differentiation (10/41; 24%), lympho-vascular invasion (11/41; 27%), muscularis mucosa invasion or deep sub-mucosal invasion (38/41; 93%). Thirteen patients (13/41; 32%) were closely monitored, and 28 (28/41; 68%) were treated by chemoradiotherapy or radiotherapy alone. In the close follow-up group, DFS, OS and CSS were 92%, 92% and 100%, respectively vs. 75%, 79% and 96%, respectively in the chemoradiotherapy group at the end of the follow-up. Serious adverse events related to chemoradiotherapy occurred in 10% of the patients. There were no treatment-related deaths. *Conclusions*: Our study shows that close follow-up may be an alternative to systematic esophagectomy after endoscopic resection of early esophageal cancer with a predicted high risk of lymph node involvement.

## 1. Introduction

Esophageal cancer is the sixth leading cause of cancer deaths, with an increasing prevalence worldwide [1]. The proportion of early (T1) stages has increased through improved endoscopic detection, and endoscopic resection of these tumors performed in expert endoscopy centers is the first step of patient management [2,3]. Endoscopic resection is curative in most intramucosal (T1a) cancers, and a subset of cancers invading the submucosa (T1b) [4].

Endoscopic resection of an early esophageal cancer can be “non-curative” in case of positive deep resection margins, or “potentially curative” but with a predicted high risk of lymph node involvement, based on the histologic parameters of the resected specimen. These histologic parameters are the invasion of the muscularis mucosae or the submucosa for squamous cell carcinoma (SCC), the invasion of the deep submucosa (>500 µm) for adenocarcinomas (ADC), or pejorative histoprognostic factors such as a poor tumor differentiation grade or the presence of lymphovascular invasion. Indeed, the risk of lymph node involvement in these cases reaches 10–20%. However, esophagectomy with lymph node dissection is indicated indistinctly in patients with a “non-curative” or “potentially curative” endoscopic resection [4]. However, esophagectomy results in 5–10% mortality according to the center’s experience, in cases of upfront resection or following initial endoscopic resection [5,6]. Furthermore, adjuvant chemoradiotherapy (CRT) after endoscopic resection of an early SCC is as effective as surgery to treat the predicted nodal disease in a recent, large prospective study [7]. In this context, some patients will not undergo surgery after endoscopic resection of a T1 esophageal cancer associated with a predicted high risk of lymph node involvement, because of major comorbidities contraindicating esophagectomy or patient refusal. These patients will be offered adjuvant CRT, or simple follow-up when adjuvant CRT is contraindicated or refused by the patient.

The objective of our study was to analyze the outcomes of non-surgical, organ preserving strategy, including adjuvant CRT or close follow-up, after the endoscopic resection of an early esophageal cancer with a predicted high risk of lymph node invasion.

## 2. Patients and Methods

### 2.1. Patients

This retrospective study was performed at two French tertiary referral centers from 2015 to 2020. All patients had an early esophageal cancer resected endoscopically with complete endoscopic and histologic (R0) resection of the tumor. The patients’ management after endoscopic resection was decided by a dedicated multidisciplinary meeting involving pathologists, gastrointestinal endoscopists and oncologists and radiotherapists, radiologists and digestive surgeons [8]. Adjuvant CRT was indicated in case of surgical contraindications, close follow-up in case of radiotherapy contraindications, or patients’ refusal of any adjuvant treatment following endoscopic resection. Data were collected from the patients’ medical files. All patients provided written informed consent to endoscopic procedures, analysis and publication of their data. The study protocol conforms to the ethical guidelines of the 1975 Declaration of Helsinki and has been approved by the ethical review committee for publications of the Cochin University Hospital (CLEP Decision N°: AAA-2020-08004).

### 2.2. Endoscopic Resections

All resections were performed *en bloc* by experienced endoscopists with patients under general anesthesia. Endoscopic mucosal resections were performed for lesions <15 mm and endoscopic submucosal dissection for lesions >15 mm in size or in case of protruding or depressed features contraindicating endoscopic mucosal resection with a cap or a ligation device.

### 2.3. Histological Analysis and Definitions

Resected specimens were pinned on polystyrene boards and fixed in 10% formalin for 24 h. After fixation, specimens were cut into 2–3 mm slices and embedded in paraffin. Blocks were further sliced at 4 μm thickness and stained with hematoxylin-eosin-saffron. Slides were analyzed by expert pathologists with extensive experience in digestive pathology and endoscopic resection specimen. Resected specimens were assessed for histological type, grade of differentiation, invasion of the lateral or deep margins, deepest tumor extension (mucosal or submucosal invasion), and presence of lymphovascular involvement (LVI). In case of submucosal invasion, the depth of tumor invasion was measured from the muscularis mucosae. Patients with curative endoscopic resection, defined by a complete endoscopic and histological resection of a well differentiated tumor without LVI and deepest invasion in the lamina propria for SCC or <500 µm in the submucosa for the ADC, were excluded from the study. Patients with non-curative endoscopic resection defined by cancer-invaded deep margins were also excluded from the study. Patients with a histologically complete resection and a high risk of lymph node involvement, defined by a poorly-differentiated tumor, LVI and/or deep mural invasion (muscularis mucosae or deeper for SCC and >500 µm submucosal invasion for ADC) were included [4,8].

### 2.4. Organ Preservation Strategy

In the follow-up group, patients were followed-up with physical examination, blood tests, upper endoscopy and chest-abdomen-pelvis computed tomography (CT) examination every three months for the first year and every six months until five years. Local recurrence was defined by histopathological analysis of esophageal biopsy specimens showing cancer cells, on the tumor resection site or in another part of the esophagus (metachronous recurrence). Distant recurrence was defined by the occurence of suspicious periesophageal lymphadenopathies or solid organ metastases. ^18FDG^positron emission tomography–CT (PET/CT) examination was only performed in patients with suspected recurrence on CT examination.

In the CRT group, patients were addressed to a radiation center. They were treated by association of chemotherapy and radiotherapy or radiotherapy alone in case of poor performance status, advanced age or major comorbid conditions, according to the radiotherapist choice. All patients received three-dimensional (3D) conformal radiation therapy to a target volume including the resection bed with a 5 cm vertical margin. Prescribed dose was 5000 cGy in daily 200 cGy/fractions, given five days a week, as in the Herskovic trial [9]. The following dose constraints were used: normal lung tissue 2 cm beyond the target volume was allowed to receive no more than 2500 cGy. The maximal dose to the heart as a whole was limited to 4000 cGy, but a dose as high as 4500 cGy could be given to less than 50% of the heart. Additional chemotherapy protocols were defined by the radiotherapist for each patient. Acute adverse events were evaluated using the National Cancer Institute Common Terminology Criteria for Adverse Events 5.0. After treatment, patients were followed-up with physical examination, blood tests, upper endoscopy and chest-abdomen-pelvis CT every three months for the first year and every six months until five years.

### 2.5. Statistical Analysis

The main outcome measurement was disease-free survival (DFS). Secondary outcome measurements were overall survival (OS), cancer-specific survival (CSS) and toxicity of chemo-radiotherapy. Data were presented as medians (ranges) and percentages.

DFS was calculated from the date of endoscopic resection to the date of (distant) recurrence or death from any cause. OS was calculated from the date of endoscopic resection to the date of death from any cause. CSS was measured from the date of endoscopic resection to the date of death from cancer. DFSs were compared using the log-rank test. All statistical analyses were performed using the R Studio statistical software (version 3.4.4).

## 3. Results

### 3.1. Patient Characteristics

Forty-one patients (36 with SCC and 5 with ADC) were included. Patients’ characteristics are detailed in Table 1. The reasons to adopt a non-surgical strategy were: history of cancer (mainly ear, nose and throat cancer) treated by radiotherapy in 14/41 (34%) patients, liver cirrhosis in 15/41 (36%) patients, respiratory insufficiency in 9/41 (22%) patients, severe cardiovascular disease in 6/41 (15%) patients, advanced age (>75 years) in 5/41 (12%) patients, patient refusal in 3/41 (7%) and other in 3/41 (7%) patients (endoscopist’s choice, vascular malformation contraindicating surgery).

### 3.2. Lesion Characteristics

The reasons for considering the lesions at high risk of lymph node involvement after endoscopic resection were: Poorly differentiated tumor (10/41, 24%), presence of LVI (11/41, 27%), SCC invading the muscularis mucosae (T1am3) or the submucosal layer in all SCC tumors (*n* = 36), ADC invading the submucosal layer beyond 500μm (*n* = 2). Among the 36 SCC, 7/36 (19%) tumors were poorly differentiated; LVI was observed in 9/36 (25%) tumors; the depth of tumor invasion was T1am3 in 7/36 (19%) tumors, superficial submucosal invasion (<200 µm) in 4/36 (11%) tumors, and deep submucosal invasion (>200 µm) in 25/36 (70%) tumors. Among the five ADC cases, 3/5 (60%) tumors were poorly differentiated; LVI was observed in 2/5 (40%) tumors; and deep submucosal invasion (>500 µm) was observed in 2/5 (40%) tumors.

### 3.3. Organ Preservation Strategy

**Figure 1:**
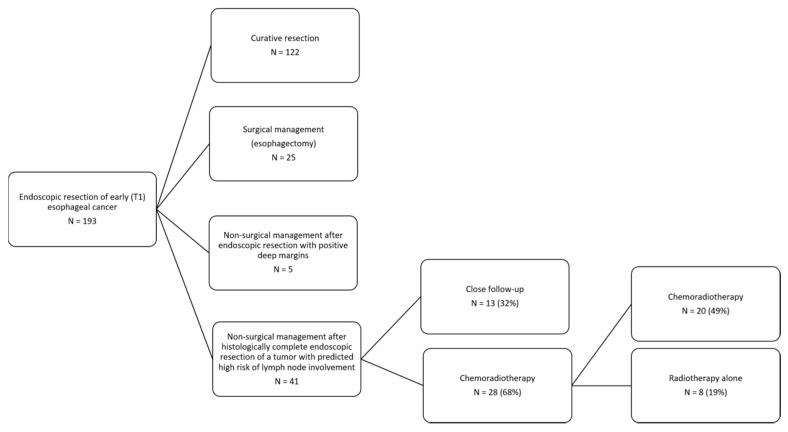
Patients flowchart.

The study flow diagram is shown in Figure 1. No further treatment and close follow-up were performed in 13 patients (32%). Twenty (49%) and eight (19%) patients were treated by CRT and radiotherapy alone, respectively.

In the close follow-up arm, there were 13 patients with 10 SCCs and three ADCs. Histological characteristics of the patients are presented in Table 2. Three tumors (3/13; 23%) exhibited more than one poor histoprognostic factor. In the CRT arm, there were 28 patients with 26 SCCs and two ADCs. Among these patients, five had poorly differentiated tumors; LVI was observed in eight patients; and the depth of tumor invasion in SCC was: T1a m3 in one patient, superficial submucosal invasion (<200 µm) in three patients, and deep submucosal invasion (>200 µm) in 22 patients. Deep submucosal invasion (>500 µm) in ADC was observed in all patients of this group. Twelve tumors (12/28; 43%) exhibited more than one poor histoprognostic factor.

In the chemoradiotherapy arm, the total radiation dose received ranged from 41 to 64 Gy on the tumor bed and lymph node areas, during 33 to 66 days, associated with 5-fluorouracil (5FU) only, 5FU-oxaliplatin or 5FU-cisplatin. Median time between the endoscopic resection and chemoradiotherapy was 2.5 months. One patient did not receive a complete treatment in the context of a poor performance status under treatment.

### 3.4. Oncological Outcomes

**Figure 2:**
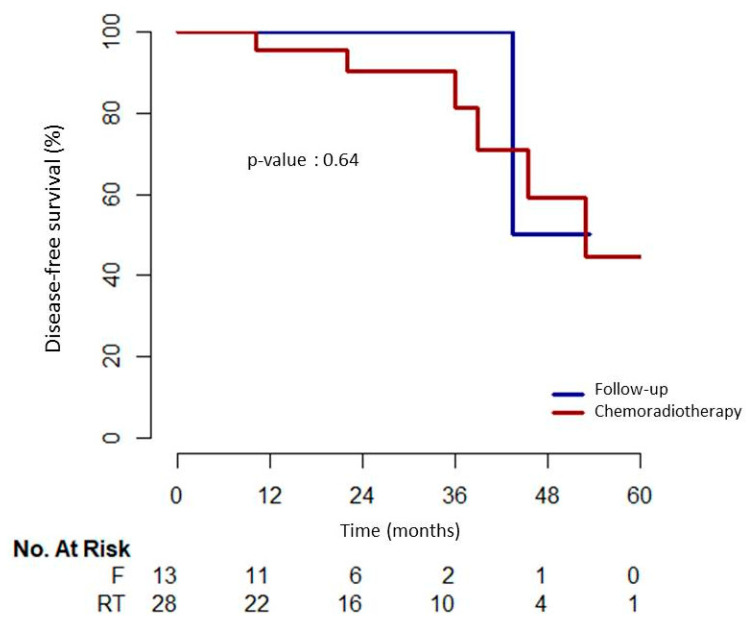
Disease free survival in the chemoradiotherapy and follow-up groups.

Only one local recurrence was observed in the CRT group, managed by a salvage surgery, without recurrence at the end of the follow-up. The patient developed recurrence 53 months after endoscopic resection of a SCC with 180 µm submucosal invasion, unamenable to endoscopic therapy. There were seven deaths during the follow-up, all non-cancer related and attributable to the patients’ comorbidities (six in the CRT group: other cancer for three patients, heart failure for two patients, and sepsis for one patient and one in the close-follow-up group: cirrhosis). At the end of the follow-up, DFS rate was 80.5% with a median follow-up (range) of 27 (3–71) months. DFS rate was 92% (12/13) in the close follow-up group after a median (range) follow-up of 19 (9–53) months, and 75% (21/28) in the CRT group, after a median [range] follow-up of 28 (3–71) months. DFS rates were 100% (13/13) and 93% (26/28) in the close-follow-up and CRT groups, respectively, both at 1 and 2 years. There was no difference between the two groups (*p* = 0.85, Figure 2). OS rate was 83%: 92% (12/13) in the close follow-up group and 79% (22/28) in the CRT group. CSS rate was 98% (40/41): 100% (13/13) in the close follow-up group and 96% (27/28) in the CRT group.

### 3.5. Toxicity of Chemoradiotherapy

Acute serious adverse events (grades 3 and 4) related to CRT occurred in 4/10 (10%) patients and were radiation esophagitis and hematological toxicity (neutropenia and febrile aplasia). No patients experienced radiation pneumonitis or pericardial effusion. There were no treatment-related deaths.

## 4. Discussion

Esophagectomy is still recommended by Japanese and European Guidelines [4,10] in case of non-curative endoscopic resection, and after resection of an early esophageal cancer considered at high risk of lymph node involvement. We have previously reported that esophagectomy after endoscopic resection allows to resect advanced residual cancer in 13% of patients and lymph node metastases in only 7% of patients, at the cost of 7% perioperative mortality and 43% severe morbidity [6]. In other studies in the field, the presence of lymph node metastases in the esophagectomy specimens ranged from 0 to 30%, with 0–14% perioperative mortality and 26–34% severe morbidity [9,10,11,12,13,14,15,16]. These figures indicate that patient selection for surgery after endoscopic resection of an early esophageal cancer is currently suboptimal [17,18]. Most importantly, esophagectomy with lymphadenectomy does not prevent late tumor recurrences, and 5-year survival after esophagectomy for T1N1 esophageal cancer does not exceed 40% [19,20]. Therefore, organ preservation strategies in the management of patients with early esophageal cancer, already performed in daily clinical practice for patients refusing or unfit for surgery, need to be evaluated before they can be proposed to patients as an alternative to esophagectomy.

In this study, an organ preservation strategy, based on CRT or close follow-up after non-curative endoscopic resection, provided high survival rates, with a DFS, OS and CSS of 80.5%, 83% and 98% respectively. More precisely, DFS, OS and CSS were 92%, 92% and 100%, respectively, in the close follow-up arm and 75%, 79% and 96% respectively in the CRT arm. The seven deaths during the follow-up were not related to esophageal cancer. Furthermore, only 10% of serious adverse events (radio-esophagitis and hematological toxicity grade 3 or 4) occurred in the CRT arm. There were no treatment-related deaths. These results suggest the efficacy and safety of close follow-up or CRT for patient with SCC and ADC with suspected high risk of lymph node involvement after histologically complete endoscopic resection.

Several retrospective studies reported the outcomes of CRT after non-curative or potentially curative endoscopic resection of SCC [12,16,21,22,23,24,25,26,27]. In these cohorts, DFS and OS ranged from 72.7 to 100% and 67.1 to 100%, respectively. Severe adverse events (mainly hematological and esophagitis or esophageal stricture) occurred in less than 25% of patients. A large prospective multicenter Japanese study included 83 patients treated with adjuvant CRT (41.4 Gy, 5FU-Cisplatin) after complete endoscopic resection of an SCC with suspected high risk of lymph node involvement, and observed a 88% DFS at 3 years, with grade 3–4 acute adverse events in 22.9% (neutropenia) and 4.2% (esophagitis) respectively, and no treatment-related death [7]. The results of these studies, summarized in Table 3 [7,12,16,21,22,23,24,25,26,27], are similar to ours. These data are limited to patients with SCC and lack a prospective comparison with esophagectomy. However, they indicate that CRT is a promising approach with acceptable results and less treatment toxicity than surgery, particularly for patients with a poor performance status. Berger et al. reported a reduced risk of cancer recurrence with CRT in a retrospective European multicenter study [28]. However, in this work, the majority of the patients had a curative endoscopic resection, the esophageal lesions were exclusively SCC, and the survival data were not available for patients with chemoradiotherapy after non-curative resection.

Yamashina et al. reported the long-term outcomes after follow-up in patients with endoscopic resection of superficial esophageal SCC [29]. In this study, the 5-year CSS was 98 and 85.7% for T1a m3 and T1b sm respectively. These results are similar to ours.

To our knowledge, our study is the first to include patients with ADC. First, patients with ADC typically have less cardiovascular or respiratory comorbid conditions compared with patients with SCC and are more frequently eligible to surgery. Second, a gastrectomy extended to the lower esophagus represents a low morbidity alternative to transthoracic esophagectomy in patients with cancers involving the esophagogastric junction. Finally, the limited effectiveness of CRT on ADC compared to SCC, as evidenced in neoadjuvant CRT trials [30,31], did not lead gastroenterologists to consider CRT in patients with ADC and a predicted high risk of lymph node involvement after endoscopic resection. Finally, several retrospective studies have suggested an extremely low risk of lymph node metastases after endoscopic resection of early esophageal ADC predicted at high risk of lymph node involvement with the current criteria [32,33,34]. Indeed, the presence of deep submucosal invasion, regarded as the main indication for esophagectomy after endoscopic resection, is actually the weakest predictor of the risk of lymph node involvement, compared to the presence of lymphovascular invasion of poor tumor differentiation [35,36].

The limitations of our work include its retrospective design, a short follow-up, and a small number of patients. The two histological types of tumor have been included, and they cannot be compared given the small number of ADC. Furthermore, the two groups were heterogeneous in terms of histoprognostic factors with a better prognosis in the close follow-up group. Indeed, tumors exhibited more than one poor histoprognostic factor in 23% of patients in the follow-up group vs. 43% in the chemoradiotherapy group. Finally, the diagnostic modalities of the close follow-up group are not currently consensual: a routine use of the PET scan would increase the sensitivity in the detection of lymph node recurrences.

Our data suggest that CRT had no added benefit over surveillance alone in terms of disease-free or cancer specific survival. Indeed, chemotherapy is poorly effective on esophageal neoplasms, and radiotherapy lacks a target lesion in these patients. Therefore, patients fit for surgery may undergo salvage surgery or CRT in case of cancer recurrence, and patients unfit for surgery are likely to die from a non-cancer related death during follow-up, as observed in our cohort. The absence of randomization, the relatively small number of patients, and the rarity of cancer recurrence do not allow to make definitive conclusions. However, considering the small number of patients included over a long period in two referral centers and the limited predicted difference in outcomes between the two strategies, an adequately powered randomized study for each cancer subtype will be extremely difficult to conduct.

## 5. Conclusions

Our study confirms that current criteria to estimate the risk of lymph node involvement after endoscopic resection of an early esophageal cancer are too stringent, and lead to perform unnecessary esophagectomies. After endoscopically and histologically complete endoscopic resection of a T1 esophageal cancer considered at high risk of lymph node metastasis, organ preservation strategies such as chemoradiotherapy or close monitoring are feasible and safe, and could constitute an alternative to systematic esophagectomy. 

## Figures and Tables

**Table 1:** Patients characteristics.

	Overall *n* = 41	Chemoradiotherapy *n* = 28	Follow up *n* = 13	*p*-Value
Age, years (median (range))	65 (47–85)	65 (53–85)	61 (47–78)	0.60
Sex ratio male/female-*n* (%)	36/5 (88%/12%)	25/3 (89%/11%)	11/2 (85%/15%)	0.64
Tumor location-*n* (%)				
Upper esophagus	5 (12%)	5 (18%)	0	-
Mid esophagus	23 (56%)	17 (61%)	6 (46%)	0.50
Lower esophagus	13 (32%)	6 (21%)	7 (54%)	0.07
Histological type				
Squamous cell carcinoma	36 (88%)	26 (93%)	10 (77%)	0.30
Adenocarcinoma	5 (12%)	2 (7%)	3 (23%)	0.30
Pejorative criteria-*n* (%)				
Deep invasion	38 (93%)	28 (100%)	10 (77%)	0.03
Poor tumor differentiation	10 (24%)	5 (18%)	5 (38%)	0.24
Lymphovascular invasion	11 (27%)	8 (28%)	3 (23%)	1
Endoscopic resection				
Endoscopic mucosal resection	7 (17%)	7 (25%)	0	-
Endoscopic submucosal dissection	34 (83%)	21 (75%)	13 (100%)	0.07

**Table 2:** Histological characteristics of the lesions in the 13 patients of the close follow-up group.

Patient	Histological Type	Quantitative Criteria		Qualitative Criteria		Number of Pejorative Criteria
		Invasion	Depth of Submucosal Invasion (µm)	Poor Tumor Differentiation	Lymphovascular Invasion	
1	SCC	T1a m3				1
2	SCC	T1b	1070	+	+	3
3	SCC	T1a m3			+	2
4	SCC	T1b	1000			1
5	SCC	T1a m3				1
6	SCC	T1a m3				1
7	SCC	T1a m3				1
8	SCC	T1a m3				1
9	ADC	T1b	110	+		1
10	SCC	T1b	2500	+	+	3
11	ADC	T1a m4		+		1
12	SCC	T1b	120			1
13	ADC	T1a m3		+		1

SCC: squamous cell carcinoma; ADC: adenocarcinoma; T1am3 or T1am4: cancer invading the muscularis mucosae; T1b: cancer invading the submucosa.

**Table 3:** Studies reporting on outcomes of chemoradiotherapy after non-curative endoscopic resection of early esophageal cancer.

Study	Patients (*n*)	SCC (*n*)	ADC (*n*)	Histologic Characteristic	Protocol	Follow-up, Median	DFS	OS	Toxicities: Grade 3–4 Adverse Events
Shimizu et al., 2004 [21]	16	16	0	T1a m3 or T1b	40 to 46 Gy 5FU-Cisplatin	39 months	100%	100% at 5 years	Hematological 12.5%
Canard et al., 2011 [22]	6	6	0	T1a m3 or T1b	NA	44 months	100%	NA	0%
Mochizuki et al., 2011 [23]	14	14	0	T1a m3: 8 T1b (superficial: 67%, deep: 33%) LVI: 2	40 Gy 5FU-Cisplatin	45 months (mean)	100% (CSS)	85.7%	0%
Ikeda et al., 2015 [12]	11	11	0	Poor differentiation 9% T1b (superficial: 27%, deep: 72%) Positive deep margins: 36% LVI: 73%	41.4 to 50.4 Gy	43 months	72.7%	89% at 3 years (with adjuvant surgery or chemoradiotherapy)	9%
Kawaguchi et al., 2015 [24]	16	16	0	T1a m3 with LVI (3) or T1b (superficial: 29%, deep: 71%)	40 to 60 Gy 5FU+/− Cisplatin	3 years	93.7%	90%	Esophageal stricture 25% Leucopenia 25% Esophagitis 12.5% Nausea18.8%
Uchinami et al., 2016 [25]	45	45	0	T1a m3: 8.5%	28 to 68 Gy +/−5FU-Cisplatin (85%)	44 months	82.9% (CSS)	NA	7%
Hamada et al., 2017 [26]	66	66	0	T1a m3:27% T1b (superficial 12%, Deep 61 %) R1:8% LVI: 55%	40 to 60 Gy	51 months	88%	75% at 5 years	Hematological 20% Non-hematological 14%
Hisano et al., 2018 [27]	13	13	0	T1a m3: 6 T1b: 7 LVI: 1 Positive deep margin: 1	40 to 61.4 Gy +/− 5FU-Cisplatin S-1 (4/13)	3 years	77.8% (CSS)	67.1%	Esophageal stricture 1 Radiation pneumonitis 1 Neutropenia 2
Suzuki et al., 2018 [16]	16	16	0	T1b 75% LVI 69% Positive deep margin 12.5%	40 to 50 Gy 5FU-Cisplatin	24 months	88%	100%	Leucopenia 25% Esophagitis 6%
Minashi et al., 2019 [7]	87	87	0	T1a m3 with LVI or T1b	41.4 Gy 5FU-Cisplatin	3 years	88%	NA	Neutropenia 22.9% Esophagitis 4.2%

SCC: squamous cell carcinoma; ADC: adenocarcinoma; T1a m3: intramucosal cancer with invasion of the lesion the muscularis mucosae; T1b: cancer with submucosal invasion; LVI: lymphovascular invasion; 5FU: 5 fluorouracile; S-1: tegafur/gimeracil/oteracil potassium; DFS: disease-free survival; OS: overall survival; CSS: cancer specific survival.
